# Understanding occupational safety and health surveillance: expert consensus on components, attributes and example measures for an evaluation framework

**DOI:** 10.1186/s12889-022-12895-6

**Published:** 2022-03-14

**Authors:** Liu Yang, Adam Branscum, Laurel Kincl

**Affiliations:** College of Public Health and Human Sciences, Oregon State University, Corvallis, OR USA

**Keywords:** Occupational safety and health surveillance, Surveillance evaluation, Guidelines, Framework, Delphi technique

## Abstract

**Background:**

Occupational safety and health (OSH) surveillance systems track work-related fatalities, injuries and illnesses as well as the presence of workplace hazards and exposures to inform prevention efforts. Periodic evaluation is critical to the improvement of these systems to meet the demand for more timely, complete, accurate and efficient data processing and analysis. Despite the existence of general guidance for public health surveillance evaluation, no tailored guidance exists for evaluating OSH surveillance systems to date. This study utilized the Delphi technique to collect consensus among experts in the United States on surveillance elements (components, attributes and measures) to inform the development of a tailored evaluation framework.

**Methods:**

A Delphi study approach with three survey rounds invited an expert panel to rate and comment on potential OSH surveillance evaluation framework elements, resulting in an optimal list of elements through the panel’s consensus. Additionally, experts completed a review of OSH surveillance systems they worked with and answered questions regarding the development of an evaluation framework. Descriptive statistics of the ratings were compiled for the Delphi process. Major themes from experts’ comments were further identified using content analysis to inform contextual information underlying their choices.

**Results:**

Fifty-four potential experts across the United States were contacted to participate in the Delphi study. Ten experts began the first survey round with eight then seven experts continuing in the subsequent rounds, respectively. A total of 64 surveillance components, 31 attributes, and 116 example measures were selected into the final list through panel consensus, with 134 (63.5%) reaching high consensus. Major themes regarding current OSH surveillance focused on resources and feasibility, data collection, flexibility, and the inter-relatedness among elements.

**Conclusions:**

A Delphi process identified tailored OSH surveillance elements and major themes regarding OSH surveillance. The identified elements can serve as a preliminary guide for evaluating OSH surveillance systems. A more detailed evaluation framework is under development to incorporate these elements into a standard yet flexible approach to OSH surveillance evaluation.

**Supplementary Information:**

The online version contains supplementary material available at 10.1186/s12889-022-12895-6.

## Background

Work-related hazards and exposures affect human health and well-being globally as well as in the United States (US). Each year, there were approximately 2.8 million nonfatal occupational injuries and illnesses and more than 5000 fatal occupational injuries in the US according to the Bureau of Labor Statistics in 2019, corresponding to 2.8 cases per 100 full-time equivalent workers (FTEs) and 3.5 fatalities per 100,000 FTEs, respectively [1]. Important data on work-related fatalities, injuries and illnesses as well as the presence of workplace hazards and exposures have been systematically collected by established occupational safety and health (OSH) surveillance systems to inform prevention efforts. In most countries including the US, OSH surveillance is largely undertaken by national and state government agencies that have the legal authority to require disease and injury reporting and access to various data sources containing OSH information [2, 3]. Being formalized since 1970 in the US, OSH surveillance has undergone continuous development in both national and state levels. Major national systems for work-related injuries, illnesses, and death have been implemented, including the Bureau of Labor Statistics (BLS) national Survey of Occupational Injuries and Illnesses (SOII) and the Census of Fatal Occupational Injuries (CFOI) [4], supplemented by other programs, such as the national Adult Blood Lead Epidemiology and Surveillance System (ABLES) and the National Electronic Injury Surveillance System—Occupational Supplement (NEISS-Work) [5]. Recognizing the pivotal role of states in OSH surveillance, the National Institute for Occupational Safety and Health (NIOSH) has been collaborating and funding state agencies for OSH surveillance since 1980s. In collaboration with the Council of State and Territorial Epidemiologists (CSTE), occupational health indicators (OHIs) surveillance has been proposed as the core activity for state-based surveillance since 2000 [6]. As of 2021, a total of 26 states have established state-based OSH surveillance systems to conduct OHIs surveillance and other expanded surveillance, following guidelines on minimum and comprehensive activities recommended by the NIOSH [7].

Despite tremendous improvement in OSH surveillance methodologies and techniques in recent decades, there have been calls for more accurate and complete data as well as more timely and efficient processing from data intake to the dissemination of interpretable health information to guide better prevention intervention practices [8–10]. Under-reporting and under-estimation of occupational injuries and illnesses has continued to be a significant concern regarding OSH surveillance data reliability [11–13]. Studies have shown an under-estimation as much as 69% in national statistics of non-fatal work-related injuries and illnesses [14–16]. Gaps and challenges also exist in other aspects of OSH surveillance, including multi-source surveillance and data integration, expanding surveillance from lagging indicators (e.g., injuries and diseases) to leading indicators (e.g., workplace exposures and hazards, safety behaviors), maintaining high confidentiality and privacy standards in data reporting and sharing, and other ongoing challenges related to funding, organizational capacity and resources [8, 9, 17–19]. On the other hand, emerging modern information technologies provide new opportunities for improving health surveillance but also pose requirements for system infrastructure, staff expertise, and coordination among systems [20, 21].

Evaluation, defined as systematic collection of information about the activities, characteristics, and outcomes of a program, is critical to the implementation and continuous improvement of a surveillance system [22, 23]. Periodic evaluation identifies gaps and potential opportunities for improvement and helps to ensure that problems are being monitored efficiently and effectively and the system is of quality and useful [24]. However, surveillance evaluation can be challenging due to the lack of consistent operational definitions and detailed guidance corresponding to the different types of health surveillance. As a result, evaluations can be incomplete, incomparable and limited in their use of guiding system improvement. It can be particularly challenging to evaluate OSH surveillance systems and no tailored guidelines have been developed for this type of surveillance. The development of surveillance systems for occupational safety and health conditions in the US has historically lagged behind those for other public health conditions, and so has its evaluation practice. Compared to a large body of literature on evaluations of infectious diseases surveillance and other types of public health surveillance, there are few published evaluations on OSH surveillance [25], and thus there is limited reference for this specific type of evaluation. A tailored evaluation framework that takes into consideration characteristics of OSH surveillance systems can guide more effective and consistent OSH surveillance evaluation practice and thus contribute to the continuous development and improvement of OSH surveillance.

### Existing guidelines and frameworks

The US Centers for Disease Control and Prevention (CDC) published *Guidelines for evaluating surveillance systems* in 1988 [26], based on which the *Updated guidelines for evaluating public health surveillance systems* were further released in 2001 [24]*.* The two guidelines are probably by far the most well-known and the de facto authoritative guidelines in public health surveillance evaluation. The evaluation method established in the two guidelines, i.e., the use of attributes, such as system simplicity, flexibility, acceptability, data sensitivity and quality, to describe and assess characteristics and performance of a surveillance system, have been widely adopted in existing surveillance evaluations [27–36]. It also establishes a framework for many other surveillance evaluation guidelines and frameworks [37–43]. However, the CDC guidelines are limited in that they provide only generic recommendations and are insufficient in guiding different types of surveillance systems [36, 44–46].

A published systematic literature review reported 15 guiding approaches for public health and animal health surveillance [46]. As of 2021, we identified a total of 34 published guidelines and frameworks (Methods described in below section; Major guidelines and frameworks and brief introductions are listed in Additional File 1). Nearly 90% of them were published since 2000, indicating an increased interest in the development of surveillance evaluation guidelines. However, most of these existing guidelines and frameworks are intended to be universally applicable or geared towards communicable diseases and animal health surveillance. We found few existing guidelines focusing on occupational safety and health surveillance, except one that was developed for national registries of occupational diseases in European Union countries [47].

Further, existing guidelines and frameworks are insufficient in terms of providing a comprehensive list of attributes and evaluation questions, as well as practical guidance on selecting, defining and assessing these attributes [46]. We have reported elsewhere the results and challenges of an OSH surveillance evaluation in Oregon [36]. We found that it was difficult to find consistent definitions of attributes and evaluation questions in existing references that were appropriate for our system under evaluation. To address the lack of tailored guidelines for evaluating OSH surveillance in the US and thus help to improve the evaluation practice, we propose a guiding framework model, which should incorporate components (i.e., main functions and activities in a system) relevant to OSH surveillance systems in the US as well as a comprehensive list of attributes corresponding to these components. In addition, a list of accessible and appropriate measures adds to the practicality of the framework. This present paper describes the first step towards this end, in which a Delphi study was designed and conducted to solicit opinions and suggestions from a panel of experts to: 1) develop a comprehensive list of framework elements including surveillance components, attributes and example measures; and 2) pilot the selected elements with current OSH surveillance systems in the US. It is expected that findings of this study can improve understanding of OSH surveillance and inform the development of a tailored evaluation framework.

## Methods

Being a consensus method, the Delphi technique is commonly used in research and guidelines development. A modified Delphi study with three internet-based survey rounds and a background survey was designed following the recommendations from common Delphi theoretical frameworks and practices in healthcare and related fields [48–58]. Experts across the US were invited to rate and comment on framework elements as well as to review their respective OSH surveillance systems.

The study obtained ethics review approval by the Institutional Review Board at Oregon State University (Review#: 8797). All participants gave informed consent. The study’s experimental protocol was in accordance to guidelines and standards set forth in the Common Rule (45 CFR 46) by the Department of Health and Human Services in the US.

### Framework elements

Common surveillance components, attributes, and example measures were compiled from a thorough literature review conducted by January 2019. A combination of three methods was used to systematically search for publications on public health surveillance evaluation guiding approaches: 1) Searching three search engines/data bases: PubMed, Web of Science and Google Scholar using combination of the following key words and wildcards (*): surveillance; and evaluat* OR assess*; and occupational OR "work related" OR workplace OR worker (the set of occupation related key words was not used for Google Scholar); 2) In Google Scholar, searching all publications that cited the two guidelines published by the CDC, *Guidelines for evaluating surveillance systems* in 1988 and *Updated guidelines for evaluating public health surveillance systems* in 2001; 3) Checking references from acquired articles. The literature search yielded a total of 2,685 articles and documents, from which we identified 34 publications aiming to provide guidelines and frameworks for evaluating public health surveillance systems.

We thoroughly reviewed the 34 publications on their merit in guiding OSH surveillance evaluation based on our subject matter knowledge and experience. This led to a total of 21 guidelines and frameworks listed in Additional File 1. During the review, we also abstracted surveillance components and attributes and their definitions, as well as example evaluation measures that are deemed to be relevant to OSH surveillance. The abstracted elements were further refined to unify terms with similar connotations, and then grouped into surveillance components and sub-components, with attributes and measures matched to specific components.

**Fig. 1:**
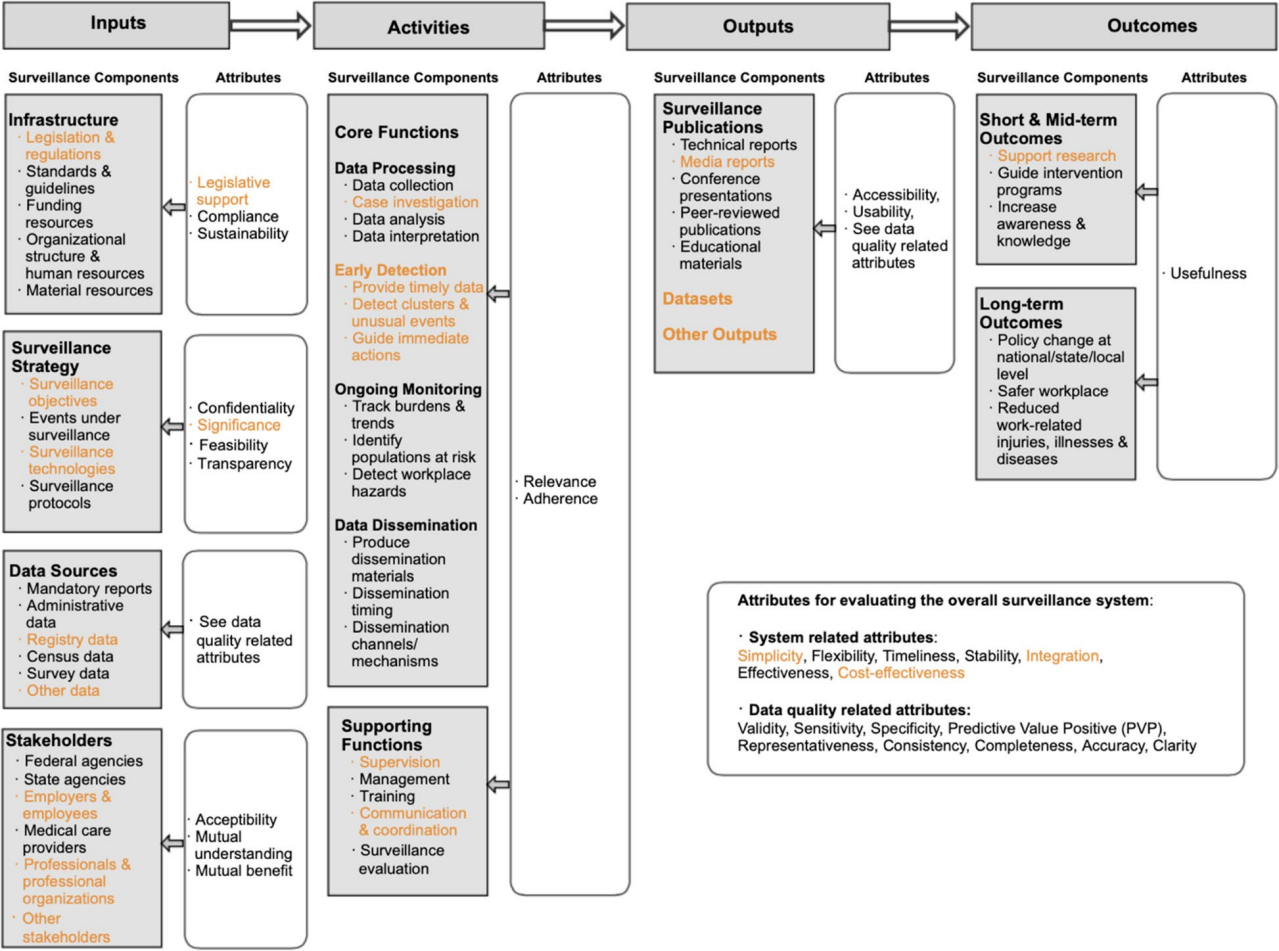
Selected components and attributes of OSH surveillance in logic model (Elements marked orange were selected with low consensus)

A finalized list contains a total of 59 surveillance components (including 47 sub-components), 32 attributes and 133 example measures. These elements were organized into a logic model that was provided to the expert panel to review and amend (See Fig. 1 for the logic model showing the selected components and attributes from the Delphi process). For example, “Infrastructure” was proposed as a component in “Inputs” of the initial logic model, with four sub-components, “Legislation and regulations”, “Funding mechanism”, “Organizational structure”, and “Resources. Three attributes were proposed to assess the “Infrastructure” component, including “Legislative support”, “Compliance” and “Sustainability”. Multiple measures were suggested for each attribute. Examples include, “Are there mandatory requirements on the establishment of the system?”, “Is the system in compliance with all legal and regulatory requirements?”, and “Is funding secure for short-term and long-term future?”.

### Delphi panel composition

The panel included experts with experience in at least one of the following two main specialty areas: 1) the operation of OSH surveillance systems in the US and, 2) evaluation of OSH surveillance systems. Potential participants were identified through multiple channels: 1) websites on national and state level OSH surveillance systems in the US; 2) authors of peer-reviewed publications of OSH surveillance evaluation; 3) recommendations from professionals in OSH surveillance; and 4) participants in the CSTE Occupational Health meeting on December 2018.

Recruitment emails were sent to 54 identified persons from 27 national and state level OSH surveillance systems in the US, and authors and researchers in OSH surveillance seeking their participation and/or their recommendation of possible participants. Considering the scope of study and resources available, we aimed for an expert panel size of 10–20 participants. Literature supports that a group of 5–10 experts would be sufficient for a heterogeneous panel, while reasonable results can be obtained with 10–15 experts in a homogenous group [59, 60]. A total of 14 people expressed interest and were invited into the first Delphi round.

Six criteria (Table 1) were evaluated for each potential panelist. A panelist had to satisfy at least three criteria to qualify as an “expert”.

**Table 1:** Preset Criteria for Experts in the Delphi Study

No	Criteria	Description
1	OSH surveillance experience	More than one year’s experience working as a key staff in OSH surveillance systems in the U.S
2	Evaluation experience	Experience in evaluating OSH surveillance systems
3	Education	Bachelor’s degree or higher in public health, safety, or related fields
4	Publications	More than three peer-reviewed publications/book or book chapter on occupational safety and health topics in the past five years, among which at least one focusing on OSH surveillance
5	Conference presentations	More than three national level conference presentations on occupational safety and health topics in the past five years, among which at least one focusing on OSH surveillance
6	Professional involvement	Current member or committee in professional organizations of occupational safety and health, public health surveillance, or program evaluation

### Survey rounds

Three rounds of internet-based surveys were administered from March 2019 to August 2019. In each round, an invitation email was sent to panelists who had completed the previous round, followed by at least two reminder emails. Panelists completed each round in 2 to 4 weeks. The first two rounds were to review 1) surveillance components and attributes, and 2) example measures, respectively. Panelists rated these framework elements on a 5-point Likert scale based on their relevance to OSH surveillance in the US and its evaluation, with 1 indicating the least relevant and 5 the most relevant. They also provided justifications, suggested revisions, additional elements, and other relevant thoughts. Statistics of the ratings and qualitative summaries for each element along with the panelist’s own rating in the first review were sent back to each panelist for a second review in the next round, where the panelist had the opportunity to reconsider his/her ratings for elements that did not have a high panel consensus. In the third round, the panelists also gave a brief review of the OSH surveillance systems they work with and answered questions regarding their system’s operation and evaluation, including strengths and weaknesses. Using selected attributes from the previous two rounds, the panelists scored their system’s performance on a scale from 0 (worst performance) to 10 (best performance) (based upon their best understanding of the systems without collecting actual evaluation evidence). Questions regarding the development of the framework were included in each round. A background survey to collect panelists’ educational and professional experience was completed between the first and the second rounds.

### Data analysis and selection of elements

Mean and mode (the most frequent rating) are common statistics used in Delphi process to measure central tendency of panelists’ ratings, or “average group estimate”. While difference between the highest and lowest ratings, SD (standard deviation of ratings), and the percent of experts giving a certain rating or selecting a certain category measure the divergence of group opinions, or the amount of panel disagreement [57, 61, 62]. For each element, quantitative statistics reflecting panel opinions were calculated. The panelist’s comments were taken into consideration to edit the element.

The following predetermined consensus criteria selected elements into three groups:

1. High consensus: mean rating and mode ≥ 4, 80% or more panelists rated 3 or higher, difference of panel ratings ≤ 2 (e.g., range from 3 to 5), and no significant edits based on panelists’ comments;
2. Low consensus/Selected for further confirmation: mean rating and mode ≥ 3 and < 4, 60% to 80% panelists rated 3 or higher;
3. Dropped: mean rating < 3, or mode < 3, or less than 60% panelists rated the element 3 or higher.

All other questions were analyzed using descriptive statistics including mean and percentage. Content analysis was performed for all comments on the elements and responses to open-ended questions in each round to identify major themes regarding the current status of OSH surveillance systems and their evaluation. Data were initially processed using Microsoft Excel. Quantitative analyses were conducted using R (version 3.6.1). Qualitative analysis was conducted using NVivo 12.

## Results

### Delphi panel participation and composition

Of the 14 people who expressed interest, 10 completed the first round survey and were included in the Delphi panel. Eight out of the ten panelists completed the second round and seven completed the third round. The initial 10 panelists were geographically distributed across the US, representing 12 states covering all five regions in the US. Of the 10 participants, seven worked for state health departments and three were from an academic setting. The panelists’ experience working with OSH surveillance systems in the US ranged from 2 to 5 years, with a mean of 4.3 years. Eight of the 10 panelists had experience with OSH surveillance evaluation. All panelists held a master’s degree or higher and had peer-reviewed publications and/or conference presentations relevant to OSH surveillance. All panel members were qualified to be experts based on the preset criteria (Table 1).

### Consensus and opinion convergence

The number of elements selected and consensus statistics from the Delphi rounds are presented in Table 2. The process resulted in 64 components (including 50 sub-components), 31 attributes, and 116 example measures in a final list, with 134 (63.5%) reaching high consensus. In general, components and attributes received higher mean rating and mode as well as smaller difference and SD than example measures. Compared to elements with low consensus, elements that received high consensus tended to have much higher mean and mode, and much smaller difference and SD, with more than 98% of panel experts rating them as $\ge$ 3.

**Table 2:** Summary statistics for framework elements (average (range))^a^

**Element**	**Delphi process**^b^	**Category**	**#Element**	**Mean rating**	**Mode**	**Difference**	**SD**	**%Panelists rated 3 or above**
**Component**	After 1st review	All	59	4.3 (3.4, 4.9)	4.6 (3, 5)	2.0 (1, 3)	0.7 (0.3, 1.2)	95 (80, 100)
		High consensus	34	4.6 (4.0, 4.9)	4.9 (4, 5)	1.6 (1, 2)	0.6 (0.3, 0.9)	99 (90, 100)
		For confirmation	22	3.9 (3.4, 4.5)	4.1 (3, 5)	2.6 (1, 3)	0.9 (0.5, 1.2)	90 (80, 100)
		Dropped	3	4.1 (3.7, 4.6)	4.3 (4, 5)	2.0 (2, 2)	0.7 (0.7, 0.7)	97 (90, 100)
	After 2nd review	High consensus	45	4.5 (4.0, 4.9)	4.9 (4, 5)	1.6 (1, 2)	0.6 (0.3, 0.9)	98 (80, 100)
		Low consensus	19	3.8 (3.5, 4.4)	4.1 (3, 5)	2.7 (2, 3)	0.9 (0.6, 1.2)	88 (60, 100)
		Dropped	1	4	5	3	1.3	50
	Final list	All	64	4.3 (3.5, 4.9)	4.6 (3, 5)	2.0 (1, 3)	0.7 (0.3, 1.2)	95 (60, 90)
**Attribute**	After 1st review	All	32	4.2 (3.6, 4.8)	4.5 (3, 5)	2.0 (1, 3)	0.8 (0.4, 1.2)	96 (80, 100)
		High consensus	24	4.4 (4.0, 4.8)	4.6 (4, 5)	1.7 (1, 2)	0.7 (0.4, 0.9)	99 (90, 100)
		For confirmation	8	3.9 (3.6, 4.3)	4.1 (3, 5)	2.8 (2, 3)	1.0 (0.7, 1.2)	89 (80, 100)
		Dropped	0	/	/	/	/	/
	After 2nd review	High consensus	26	4.4 (4.0, 4.8)	4.6 (4, 5)	1.7 (1, 2)	0.7 (0.4, 0.9)	99 (90, 100)
		Low consensus	5	3.7 (3.3, 4.0)	3.6 (3, 5)	2.8 (2, 3)	0.9 (0.7, 1.1)	90 (80, 100)
		Dropped	1	4.1	5	2	0.9	90
	Final list	All	31	4.3 (3.3, 4.8)	4.5 (3, 5)	1.9 (1, 3)	0.7 (0.4, 1.1)	98 (80, 100)
**Measure**	After 1st review	All	133	3.8 (2.5, 4.8)	4.0 (2, 5)	2.3 (1, 4)	0.9 (0.4, 1.4)	92 (50, 100)
		High consensus	55	4.2 (4.0, 4.8)	4.6 (4, 5)	1.9 (1, 2)	0.7 (0.5, 1.0)	99.8 (88, 100)
		For confirmation	71	3.6 (3.0, 4.3)	3.6 (3, 5)	2.5 (1, 4)	0.9 (0.4, 1.4)	89 (63, 100)
		Dropped	7	3.0 (2.5, 3.8)	2.9 (2, 4)	2.9 (2, 3)	1.1 (0.9, 1.3)	66 (50, 88)
	After 2nd review	High consensus	63	4.2 (4.0, 4.8)	4.6 (4, 5)	1.9 (1, 2)	0.7 (0.5, 1.0)	99.8 (88, 100)
		Low consensus	53	3.5 (3.0, 4.1)	3.5 (3, 5)	1.8 (0, 4)	0.7 (0.0, 1.2)	94 (75, 100)
		Dropped	10	2.8 (2.6, 3.1)	2.9 (2, 3)	2.5 (2, 3)	0.8 (0.7, 1.1)	69 (63, 88)
	Final list	All	116	3.9 (3.0, 4.8)	4.1 (3, 5)	1.9 (0, 4)	0.7 (0.0, 1.2)	97 (75, 100)

^a^Elements were rated in 5-point Likert scale, with 1 indicating the least relevant and 5 the most relevant

^b^Components and attributes were reviewed in the first and second rounds; example measures were reviewed in the second and third round

Changes of statistics through rounds were further checked to investigate opinion convergence and the panel agreement. The SD and difference of elements rated in the second review reduced (Wilcoxon signed rank test *P*-values < 0.05), indicating increased opinion convergence. More elements had small difference in the final list compared to the initial list (data not shown). For example, the percentage of measures with a difference of 2 or below increased from 66.9% to 92.2% in the second review.

An OSH surveillance logic model was finalized to include components and attributes that were selected after two panel reviews (Fig. 1). A complete list of all selected elements as well as their finalized description can be found in Additional File 2.

### Piloting selected elements with OSH surveillance systems

Six state-level and one national OSH surveillance systems in the US were evaluated by panelists in the last Delphi round. The majority of the systems were supported by national funds (86%). Nearly two thirds of the systems (57%) conducted more than one type of OSH surveillance. Three most common types of surveillance were the OHIs surveillance (86%), Injury surveillance (71%), and ABLES (57%). Nearly half of the systems (43%) conducted only secondary data analysis. More than 57% of the systems were never or rarely evaluated and no system was evaluated frequently. For those ever being evaluated, common purposes were to assess implementation (80%) and performance (60%). Need assessment, cost-effectiveness and data quality assessment were rarely or never conducted. Seven attributes were scored high (> 8.0) in these systems, among which, compliance and confidentiality received the highest score (9.0), followed by significance, relevance, adherence, accuracy, and clarity. Three attributes received the lowest score: legislative support (5.9), timeliness (5.7), and integration (3.9). Common perceived weaknesses included high quality data sources, being able to conduct various surveillance activities, dissemination, and creating outcomes and impacts. Major constraints included lack of resources, legislative support, and recognition and integration into the general public health domain.

### Major themes identified

Content analysis on the ample commentary from panel experts over the three Delphi rounds revealed the themes described below. Table 3 includes the theme categories and most frequent codes with associated frequency results.

**Table 3:** Major themes from experts' comments

Theme codes	Frequency
**Status quo and issues**	
Resources and feasibility	22
Rely on secondary data	10
Authority and data collection	8
Gaining buy-in	8
**Ideal OSH surveillance**	
Data collection	11
Data dissemination	9
Plan and strategy	7
**Implications for evaluation**	
Inter-relatedness among elements	55
Flexibility	25
Importance of framework	10
Stage of evaluation	8
Weights of elements	8

#### Status quo and issues

Resource constraints and feasibility were common issues noted by the panel. One expert commented that “the reality is that resources for this type of work [OSH surveillance] are historically VERY limited.” Funding was reported as not stable. A panelist wrote that it would be “best if … built into the regular funding cycle.” Technological resources and legislative supports were reported as basic or even lacking. The lack of resources included comments on the dependence of external data in current OSH surveillance. An expert commented that “without them [legislation and regulations] one may end up using existing data not designed for OSH” and another wrote that “most of the data systems in use currently for OSH surveillance were designed for an entirely separate purpose (i.e. financial tracking and reimbursement)”. Reported issues with various existing data sources included the lack of information/variables critical to OSH surveillance (e.g., industry and occupation information), and the data timeliness. A panelist commented, “Often there is a substantial lag time in the availability of surveillance data for public health actions”. A recurring message from the panel experts was to have a “realistic” expectation about current OSH surveillance.

#### Ideal OSH surveillance

Some system requirements may not seem readily feasible given limited resources and thus belong to “an ideal” OSH surveillance. Experts’ comments helped to outline an ideal OSH surveillance, in which all necessary infrastructures and resources including funding, legislation, human resources, technological platform and stakeholders’ collaboration, were well-integrated to support key surveillance activities from data collection to dissemination as well as interoperability among data systems. Strategic planning was emphasized in the ideal OSH surveillance. “Having a well thought out plan…is critical to developing a streamlined system”. Plans and protocols should cover both existing and potential surveillance activities.

#### Implications for evaluation and a framework

Nearly all experts held positive expectations about the development of a tailored framework for OSH surveillance evaluation. One expert commented that “It would be helpful to have an evaluation framework specific to OSH surveillance …” and another noted that “we've been struggling with developing a systematic evaluation approach, so it will be good to have consensus on components of an evaluation”. Experts called for flexibility in OSH surveillance evaluation, because “a ‘one-size fits all’ mentality of program evaluation is often unnecessary”, and an evaluation framework should “allow for ‘picking and choosing’ of those evaluation components”. Factors such as differences between systems, changing surveillance objectives and OSH conditions over time, and non-routine activities affect the evaluation choices. One expert commented that “… evaluating it [the infrastructure] seems difficult as it constantly changes and differs from state to state”. Another expert stated, “Case definitions are often lacking for OSH across jurisdictions, states, areas.” As to assessing the significance of surveillance, one expert wrote that “Priorities change and the most useful surveillance systems are those that have a historical trove of information about priority conditions that emerge as ‘significant concerns’ at some point.”

#### Inter-relatedness among components and attributes

Fundamental and critical components such as funding, resources, organizational structure, management and training, standards and guidelines, and strategies were commonly stated as impacting the system’s performance and stability. For example, experts commented that “Infrastructure is necessary for an effective and efficient public health surveillance system” and the surveillance strategy “impacts standardization and consistency over time and among data sources”. Some elements were regarded as more central in relation to other elements. For example, experts commented that stakeholders’ acceptance and participation were impacted by the system’s compliance, transparency, simplicity and usefulness, while they in turn impacted various aspects of the system’s performance. They stated that “without the willingness, the data is not as good”, “whether a system will function smoothly, meets the intended purpose and can be sustained also depends on its acceptability by stakeholders.” Attributes such as data quality, data timeliness and usefulness were also regarded as central elements.

## Discussion

The lack of comparability and replicability in OSH surveillance evaluations limits their usefulness. Agreed terms and their descriptions facilitate understanding of OSH surveillance and thus contribute to the improvement of surveillance and its evaluation. This three-round survey study based on the Delphi technique sought experts’ consensus on framework elements. Experts’ comments on elements as well as the current status of their respective OSH surveillance systems further informed contextual information underlying their choices.

### Surveillance elements

This study leveraged a combination of central tendency and divergence measures to differentiate the framework elements. The resulting logic model and list (Fig. 1 and Tables in Additional File 2) contain elements receiving high and low level of consensus. A key to a successful Delphi process is to allow panelists to reconsider and potentially change their opinions after referring to the views of their peers [54, 63]. The second review helped to reach panel opinion convergence for elements that the panel rated low and/or held more dispersed views for in the first review. After the second review, both the difference and SD reduced significantly. The change of ratings in this study suggested that the “group opinions” had guided the panel experts’ reconsideration of their stance. A strength in this Delphi study is that experts were provided with feedback in both quantitative and qualitative summaries, as literature has suggested that qualitative feedback is an effective way to mitigate experts’ propensity to stick to their initial opinions [64, 65].

Many elements in the “Inputs” in the logic model were considered fundamental and critical to the OSH surveillance. The need for more relevant OSH surveillance data sources and stakeholders was also identified by the panel. Regarding surveillance activities, not surprisingly, data processing, ongoing monitoring and data dissemination and their sub-components (except case investigation) were considered to be relevant to OSH surveillance. These activities are also well-accepted components of public health surveillance to date [66].

The study revealed that some components and attributes were less feasible to current OSH surveillance due partly to limited resources, for example, the component, “early detection” and its three sub-components, “provide timely data”, “detect clusters and unusual events” and “guide immediate actions”. Despite that guiding immediate action was proposed for an ideal OSH surveillance [9], the panel experts thought that these activities were less relevant to current OSH surveillance because they were “resources intensive”. Among low consensus attributes, “simplicity” is worth mentioning. In general, a surveillance system should be effective and as simple as possible [24, 67, 68]. Interestingly, the panelists had conflicting perspectives towards simplicity: OSH surveillance was fairly simple, but, on the other hand, that surveillance systems can be intricate.

Example measures received lower mean rating and mode as well as larger difference and SD than components and attributes in the Delphi process, reflecting relatively larger divergence of panel opinions. Our speculation is that components and attributes tend to appear more often in existing evaluation guidelines with relatively finite definitions and connotations, while measures, which represent the operationalization of attributes into measurable indicators, tend to be more flexible and selected/developed at the evaluator’s discretion. As such, the expert panel might hold more diversified opinions on the relevance of a measure to OSH surveillance. Since different measures may be geared towards different aspects of the attributes, the development of a standard yet flexible guiding framework can further test the comprehensiveness and applicability of selected measures, including any customization of evaluation metrics.

### Current OSH surveillance status and implications

This study showed that nearly half of the OSH surveillance systems only conducted secondary data analysis. The most common type of surveillance was the OHIs surveillance, which focuses on secondary data analyses with multiple external data sources. The findings are in line with the recent report by the National Academies of Sciences, Engineering, and Medicine that, “there is no single, comprehensive OSH surveillance system in the US, but rather an evolving set of systems using a variety of data sources that meet different objectives, each with strengths and weaknesses” [9]. Limitations with current available data sources presents challenges to OSH surveillance in the US as well as in many other countries [69]. For example, data completeness and accuracy are often compromised with a single data source due to its potential under-coverage and under-reporting issues [70]. On the other hand, data integration has been an issue for surveillance with multiple data sources. We have reported the utilization of workers’ compensation data from different sources for injury research and surveillance and found methodological challenge in developing crosswalk to communicate different data coding systems [71].Both the experts’ comments and their reviews of respective systems highlighted limited resources for OSH surveillance, which is also related to the lack of or less frequent evaluation for the systems. Experts reported barriers to evaluation including staff time and budget, as well as difficulty in reaching out to stakeholders. A major implication is the consideration of flexibility and feasibility in deciding evaluation objectives and criteria. Although feasibility is always a consideration, it seemed to be more remarkable with OSH surveillance, as the panel experts pointed out that many criteria were ideal but not readily feasible due to limited resources.

The identified network relationships among elements are similar to an existing study on animal health surveillance systems [72]. For example, the expert panel pointed that some surveillance elements, such as organization and management, resources and training were fundamental as they impacted the other elements the most, while some other elements, such as acceptability, data completeness, stability and sustainability could both be remarkably impacted and impact other elements, serving an intermediate role in the relationship network. These findings can guide the prioritization of evaluation focuses. Limited by the main focus in this study, in-depth investigation on this topic was not available. Further research is needed to comprehensively understand interactions among OSH surveillance components and attributes and to investigate weights for elements based their interactions.

This study highlighted the need for tailored guidelines for OSH surveillance evaluation. According to the panel experts’ comments in the Delphi process, they struggled with developing a systematic evaluation approach and current guidelines were insufficient to accommodate systems for non-communicable diseases and systems that rely on multiple data sources such as OSH. Relevant components, attributes and measures identified in this study can guide more relevant evaluation of OSH surveillance systems. The study team is further working on a more detailed evaluation framework based on study findings to bridge this gap (manuscript in preparation).

### Limitations

A few limitations should be recognized in this study. The study formed a Delphi panel with 10 experts in the first round. Although the panel size met our goal, it was a relatively small panel as compared to existing Delphi studies, in which the number of panelists varied from around 10 to more than 1,000 [73]. In general, the reliability of panel judgement tends to increase with the number panel experts. However, larger panel size may raise issues of manageability and attrition, which may adversely impact the study reliability and validity [62, 73]. Researchers pointed that it was likely that the Delphi study reliability would decline rapidly below six participants while the improvement of reliability would be subjected to diminishing returns after the first round with a panel of 12 panelists or more [54]. To date, there has been no consensus on Delphi panel size, and the numbers in existing studies usually varied according to the scope of the problem and resources (time and budget) available [62, 73].

Participation rate in the second and third round in this study was 80% and 70% respectively. Drop-out has been recognized as a particular challenge in Delphi process given its iterative feature which demands the panelists’ time commitment. Through our communication with the panelists who dropped out, we learned that time conflict was the main reason for them to stop. A review paper showed that response rate in the third round can be as low as 16% for large Delphi studies [49]. Despite we adopted multiple ways recommended in literature to increase returns, including seeking experts recommended from the field, frequent reminders and active contacts even after the specified deadlines [52], the drop-out in the following two rounds may more or less bias the information being gathered in this study. We had to assume that the expert(s) who dropped in the second review held the same view as in his/her first review, but this might have not been true.

Similar to any other consensus methods, the Delphi technique is subject to cognitive bias in information exchange and decision-making processes related to the participants’ backgrounds and personal traits [74]. Potential participants were identified using multiple information sources, however, despite the effort to recruit a panel representing both the national and state level OSH surveillance systems, most panel participants in this study were from state-level surveillance systems.

Researchers have warned that even the most well-planned Delphi may not yield an exhaustive nor all-inclusive set of ideas [51]. The finalized list in this study may not cover every aspect of OSH surveillance given limited existing reference for this field. With continued efforts promoting OSH surveillance and its evaluation, more elements that are relevant and practical to OSH surveillance evaluation may be added into the list.

## Conclusion

This paper described the development of a comprehensive list of components, attributes, and example measures relevant to OSH surveillance systems in the US by conducting a three-round Delphi survey study with experts across the US who had experience in OSH surveillance operation and/or with surveillance evaluation.

OSH surveillance components, attributes and example measures identified in this study can serve as a preliminary guide for evaluating OSH surveillance systems. The evaluators could choose components and attributes that are appropriate to their systems and use example measures to develop their own evaluation metrics. Future research and practice are needed to further refine and enrich the list of elements and explore its applicability to OSH surveillance evaluation. The research team is further developing a more detailed evaluation framework based on the study findings to provide standard yet flexible guidance on how to select and prioritize these elements for evaluating different types and stages of OSH surveillance systems.

## Supplementary Information

**Additional file 1: Table 1-1.** Major guidelines/frameworks referenced in this study.

**Additional file 2: Table 2-1.** OSH surveillance components selected with high/low consensus. **Table 2-2.** OSH surveillance attributes selected with high/low consensus. **Table 2-3.** Example measures selected with high/low consensus.

## Data Availability

All data generated or analyzed during this study are included in this published article and its supplementary information files.

## Abbreviations

ABLES: Adult Blood Lead Epidemiology and Surveillance System
BLS: Bureau of Labor Statistics
CDC: Centers for Disease Control and Prevention
CFOI: Census of Fatal Occupational Injuries
CSTE: Council of State and Territorial Epidemiologists
FTE: Full-Time Equivalent
NEISS-Work: National Electronic Injury Surveillance System—Occupational Supplement
NIOSH: National Institute for Occupational Safety and Health
OHIs: Occupational Health Indicators
OSH: Occupational Safety and Health
SOII: Survey of Occupational Injuries and Illnesses
