# Supplementary material for: Understanding occupational safety and health surveillance: expert consensus on components, attributes and example measures for an evaluation framework
Source: BMC Public Health. 2022 Mar 14;22:498. doi: 10.1186/s12889-022-12895-6 (PMC8922762; doi:10.1186/s12889-022-12895-6)
Supplement: Supplementary file 1 — Additional file 1: Table 1-1. Major guidelines/frameworks referenced in this study. [file 12889_2022_12895_MOESM1_ESM.docx]

# Additional File 1

**Table 1-1**: Major guidelines/frameworks referenced in this study

| **Guideline/framework** | **Brief introduction** |
| --- | --- |
| **Updated guidelines for evaluating public health surveillance systems.** Centers for Disease Control and Prevention (CDC). Updated guidelines for evaluating public health surveillance systems: recommendations from the guidelines working group. Morb Mortal Wkly Rep. 2001;50(No. 13):1-36. | This is the most well-known guideline and for all kinds of public health surveillance systems. It provides six evaluation steps, adapted from the Framework for Program Evaluation in Public Health [1]. Ten general evaluation attributes are recommended and discussed.   1. Centers for Disease Control and Prevention (CDC), Program Performance and Evaluation Office. Framework for program evaluation in public health. Morbidity and Mortality Weekly Report. 1999;48(No.RR-11):1-40. |
| **Injury Surveillance Guidelines**  Holder Y, Peden M, Krug E, et al (Eds). Injury Surveillance Guidelines. Geneva, World Health Organization; 2001. | These guidelines provide the design, implementation and evaluation of injury surveillance systems. It proposes and briefly discusses three types of evaluation for an injury surveillance system, i.e., retrospective, process and system environment evaluation. |
| **Framework and Tools for Evaluating Health Surveillance Systems** Health Canada. Framework and Tools for Evaluating Health Surveillance Systems. Canada: Population and Public Health Branch, Health Canada; 2004. | This Canadian framework provides a standard approach for evaluating public health surveillance systems. It outlines and discusses six evaluation steps. System performance characteristics and quality attributes are introduced and detailed in an appendix of glossaries and terms. |
| **Framework for evaluating public health surveillance systems for early detection of outbreaks: recommendations from the CDC Working Group.**  Centers for Disease Control and Prevention (CDC). Framework for evaluating public health surveillance systems for early detection of outbreaks: recommendations from the CDC Working Group. Morb Mortal Wkly Rep. 2004;53(No. RR-5):1-13. | This CDC guide is for the evaluation of public health outbreak surveillance systems (e.g., syndromic surveillance). It discusses four tasks in an evaluation and important attributes in each task. Emphasis is given to timeliness and the balance among other attributes (e.g. sensitivity). |
| **Communicable Disease Surveillance and Response Systems: Guide to Monitoring and Evaluating**  World Health Organization (WHO). Communicable Disease Surveillance and Response Systems: Guide to Monitoring and Evaluating. Geneva: World Health Organization (WHO); 2006. | WHO proposes a structural model for communicable diseases surveillance systems. It includes a detailed introduction on each component and attributes. It also discusses evaluation steps for two types of evaluation, i.e. routine tracking and periodic assessment. |
| **Proposal of a framework for evaluating military surveillance systems for early detection of outbreaks on duty areas**  Meynard J-B, Chaudet H, Green AD, et al. Proposal of a framework for evaluating military surveillance systems for early detection of outbreaks on duty areas. BMC Public Health. 2008;8(1):146. | This military framework is for evaluating syndromic surveillance systems. It incorporates characteristics of French and British military syndromic surveillance systems. Evaluation parameters (i.e. attributes) are specified for different types of evaluation corresponding to the development of a system (e.g., initial evaluation, final evaluation). |
| **Effective environmental public health surveillance programs: a framework for identifying and evaluating data resources and indicators**  Malecki KC, Resnick B, Burke TA. Effective environmental public health surveillance programs: a framework for identifying and evaluating data resources and indicators. J Public Health Manag Pract. 2008;14(6):543–551. | This framework is for the evaluation of surveillance data sources and indicators. It discusses different aspects to be considered in an evaluation, such as scientific relevance and feasibility of an indicator. Attributes are listed as criteria for each aspect. |
| **Assessing the National Health Information System: An Assessment Tool (v4.0)**  World Health Organization (WHO). Assessing the National Health Information System: An Assessment Tool (v4.0). Geneva: World Health Organization; 2008. | This tool aims to provide a guide for assessing national level health information systems (HIS). It specifies HIS components and standards and provides the scoring framework for each component as a practical tool. |
| **Characteristics of national registries for occupational diseases: international development and validation of an audit tool (ODIT)**  Spreeuwers D, de Boer AG, Verbeek JH, van Dijk FJ. Characteristics of national registries for occupational diseases: international development and validation of an audit tool (ODIT). BMC Health Serv Res. 2009;9:194. | Aimed to guide the evaluation of national registries of occupational diseases in EU countries. The audit tool includes indicators assessing a national registry system which include monitor and/or alert functions. |
| **The development of an evaluation framework for injury surveillance systems**  Mitchell RJ, Williamson AM, O’Connor R. The development of an evaluation framework for injury surveillance systems. BMC Public Health. 2009;9(1):260. | This describes the development of a framework for the evaluation of injury surveillance systems. Characteristics/attributes practical to injury surveillance are listed with experts’ ratings. Definitions and evaluation criteria are included. |
| **Evaluating Public Health Surveillance**  Groseclose SL, German RR, Nsubuga P. Evaluating Public Health Surveillance. In Lee LM, Teutsch SM, Thacker SB, Louis MES. Principles and Practice of Public Health Surveillance. 3 edition. Oxford; New York: Oxford University Press; 2010. | This textbook chapter explains the evaluation steps and attributes. Important concepts and considerations for each step are discussed. It includes definitions and methods for evaluating attributes. Attributes beyond the CDC updated guidelines are also discussed. Example evaluations are also provided. |
| **OASIS: an assessment tool of epidemiological surveillance systems in animal health and food safety**  Hendrikx P, Gay E, Chazel M, et al. OASIS: an assessment tool of epidemiological surveillance systems in animal health and food safety. Epidemiol Infect. 2011;139(10):1486-1496. | This is a standardized tool for assessing animal healt^1^h surveillance systems. It provides assessment criteria for different surveillance system functions and the scoring grids for each criterion. |
| **Conceptual evaluation of population health surveillance programs: method and example**  Allaki FE, Bigras-Poulin M, Ravel A. Conceptual evaluation of population health surveillance programs: method and example. Prev Vet Med. 2013;108(4):241–252. | This provides a conceptual model to evaluate a public and animal health surveillance program based on concepts underlying the surveillance program (i.e., what the program is), which are compared to the theoretical standards for health surveillance (i.e., what the program should be). Components and attributes are discussed as theoretical standards. |
| **Evaluating a National Surveillance System**  UNAIDS/WHO working group on global HIV/AIDS and STI surveillance. Evaluating a National Surveillance System. Geneva: World Health Organization; 2013. | This UN AID/WHO framework provides guidance for the evaluation of national level HIV surveillance systems. It covers various aspects in evaluating HIV/AIDS surveillance and programs, such as inventory, design and implementation, surveillance data quality. Case studies are also provided as illustrations. |
| **Evaluating an NCD-Related Surveillance System**  Centers for Disease Control and Prevention (CDC). Evaluating an NCD-Related Surveillance System. Atlanta, GA: Centers for Disease Control and Prevention; 2013. | This is a guide/tutorial for evaluating non-communicable disease (NCD) surveillance systems based on CDC’s Updated guidelines. It discusses the six steps recommended in the CDC updated guidelines. Methods and measures for evaluating attributes in the context of NCD surveillance are discussed. |
| **Animal Health Surveillance Terminology Final Report from Pre-ICAHS Workshop**  Hoinville L. Animal Health Surveillance Terminology Final Report from Pre-ICAHS Workshop. In: International Conference on Animal Health Surveillance; Vol 17. 2013:1-27. | This terminology book is tailored to animal health surveillance. Three groups of terminologies are listed and defined: general terms, characteristics of surveillance activities, attributes. |
| **The EVA Tool: An Integrated Approach for Evaluation of Animal Health Surveillance Systems. RISKSUR Project**  Calba C, Cameron A, Goutard F, et al. The EVA Tool: An Integrated Approach for Evaluation of Animal Health Surveillance Systems. RISKSUR Project; 2013. | The EVA tool provides comprehensive guidance for integrated evaluation of animal health surveillance systems including economic evaluation. It lists attributes related to the system organization, function, effectiveness, value and economic efficiency. Ranking of attributes is also considered in the tool. Example evaluation questions are provided for an evaluation. |
| **European Data Quality Monitoring and Surveillance System Evaluation Handbook** European Center for Disease Prevention and Control (ECDC). Data Quality Monitoring and Surveillance System Evaluation - A Handbook of Methods and Applications. Stockholm: ECDC: European Center for Disease Prevention and Control; 2014. | This is a guide for evaluation of public health surveillance systems in EU/EEA Member States. It includes a detailed introduction and guide on evaluating quality attributes in the context of communicable diseases surveillance. |
| **SERVAL: a new framework for the evaluation of animal health surveillance**  Drewe JA, Hoinville LJ, Cook AJC, Floyd T, Gunn G, Stärk KDC. SERVAL: a new framework for the evaluation of animal health surveillance. Transbound Emerg Dis. 2015;62(1):33-45. | This is a generic framework to assist with evaluation of animal health surveillance systems. It discusses evaluation steps and key questions related to each step. Guides on attributes and their selection are introduced with a selection matrix considering the importance of attributes to different surveillance objectives. It also includes guide on economic evaluation of the system. |
| **Brief Evaluation Protocol for Public Health Surveillance Systems**  Hoffman S, Dekraai M. Brief Evaluation Protocol for Public Health Surveillance Systems. the Public Policy Center, University of Nebraska; 2016. | This protocol provides a practical tool based on the CDC updated guidelines to assist evaluation of a public health surveillance system in a short period of time and with limited resources. Examples and working tables are included to illustrate evaluation steps and the evaluation of attributes. |
| **SurF: an innovative framework in biosecurity and animal health surveillance evaluation**  Muellner P, Watts J, Bingham P, et al. SurF: an innovative framework in biosecurity and animal health surveillance evaluation. Transbound Emerg Dis. 2018. | This proposes a generic framework to ensure consistent evaluation of New Zealand biosecurity and animal health surveillance systems. It proposes four phases/steps in an evaluation as well as activities within each phase. It suggests 29 attributes which are divided into core and accessory attributes. |
