# Supplementary material for: Understanding occupational safety and health surveillance: expert consensus on components, attributes and example measures for an evaluation framework
Source: BMC Public Health. 2022 Mar 14;22:498. doi: 10.1186/s12889-022-12895-6 (PMC8922762; doi:10.1186/s12889-022-12895-6)
Supplement: Supplementary file 2 — Additional file 2: Table 2-1. OSH surveillance components selected with high/low consensus. Table 2-2. OSH surveillance attributes selected with high/low consensus. Table 2-3. Example measures selected with high/low consensus. [file 12889_2022_12895_MOESM2_ESM.docx]

# Additional File 2: All selected elements

**Table 2-1**. OSH surveillance components selected with high/low consensus

| **Logic Model** | **Component** | **Sub-component** | **Definition** | **Mean rating** | **High Consensus** |
| --- | --- | --- | --- | --- | --- |
| Inputs | Infrastructure | / | Basic structure and foundations for establishing and maintaining a surveillance system. It should be flexible and able to change/evolve as the system grows and as needed. | 4.6 | Y |
|  |  | Legislation & regulations | Legal requirements and stipulations supporting the establishment of the surveillance activities and mandatory requirements that the system should follow. | 3.8 | N |
|  |  | Standards & guidelines | Available standards and guidelines identified to guide the development, implementation, maintenance and evaluation of the surveillance system. | 4.8 | Y |
|  |  | Funding resources | Funding sources, funds available for developing and implementing basic and advanced/expanded surveillance activities, and funding continuity. | 4.5 | Y |
|  |  | Organizational structure & human resources | Organization of the surveillance system and human resources, including the system's leadership, staff, communication and coordination mechanism. | 4.1 | Y |
|  |  | Material resources | Logistics, material items and technological resources vital to the surveillance system. | 4.6 | Y |
|  | Surveillance strategy | / | Guiding standards, methodologies and procedures for the establishment and maintenance of a surveillance system, including surveillance objectives, events under surveillance and case definitions, available data sources and surveillance techniques. | 4.9 | Y |
|  |  | Surveillance objectives | Objectives and priorities relevant to the current and emerging occupational safety and health needs should be clearly stated to provide an overall guide to the surveillance system. Objectives should be specific, adaptable to changing needs, and measurable. | 4.4 | N |
|  |  | Events under surveillance | Selection and prioritization of occupational safety and health cases/events under surveillance in accordance with the surveillance objectives. For each event, case definition is clear and valid without ambiguity. Available data sources and analysis methods are specified. | 4.2 | Y |
|  |  | Surveillance technologies | Technologies enable effective and efficient implementation of surveillance activities. Common technologies include devices for surveillance (e.g. sensors, computers, smartphones), technical platforms (e.g. electronic database for health records, online data sharing mechanism, analytical software), and surveillance techniques (e.g. standardized terminology, data encoding system, analytic techniques). | 3.7 | N |
|  |  | Surveillance protocols | Detailed protocols established to regulate surveillance activities, including the list of prioritized events under surveillance, case definitions, applicable data sources, data collection and processing methods, frequency of reports and ways for data dissemination, as well as data quality control process and periodical evaluation mechanism. | 4.8 | Y |
|  | Data sources | / | Instead of being a single, comprehensive surveillance system, current OSH surveillance systems make use of a variety of data sources which are collected by different organizations with different surveillance objectives, strengths and weaknesses. | 4.7 | Y |
|  |  | Mandatory reports | Certain diseases are required to be notified to state health departments or Poison Control Centers by health care providers, hospitals and emergency departments, clinical laboratories. The list of notifiable diseases is historically geared for infectious diseases but now include some occupational and environmental health conditions and exposures such as lead poisoning. | 4.6 | Y |
|  |  | Administrative data | Includes hospital discharge data, emergency department data, hospital outpatient data, workers' compensation data, OSHA records, Coast Guard records, etc. All payer claims data and electronic health records are also potential data sources. | 4.6 | Y |
|  |  | Registry data | Includes birth and death certificates, cancer registries, birth defect registries, trauma registries, and burn registries, etc. | 4.2 | N |
|  |  | Census data | Provides injury and illness data such as the Census of Fatal Occupational Injuries (CFOI), and demographics data such as Quarterly Census of Employment and Wages (QCEW). | 4.6 | Y |
|  |  | Survey data | Include state and national Survey of Injuries and Illnesses (SOII) data, Behavioral Risk Factor Surveillance System data (BRFSS), Youth Risk Behavior Surveillance System (YRBS), National Health Interview Survey (NHIS), National Health and Nutrition Examination Survey (NHANES), etc. | 4.2 | Y |
|  |  | Other data | Other potential data sources for OSH surveillance may include news reports, Google alerts. Social media and smart devices are promising new data sources. | 3.8 | N |
|  | Stakeholders | / | Those who provide data, funding or technical support; or are involved in the surveillance activities such as survey participants; or impacted by surveillance activities and results; or surveillance data users. | 4.4 | Y |
|  |  | Federal agencies | Various federal agencies may play a role, including the Bureau of Labor Statistics (BLS), the National Center for Health Statistics (NCHS), the Occupational Safety and Health Administration (OSHA), the Mine Safety and Health Administration (MSHA), the National Institute for Occupational Safety and Health (NIOSH). Federal agencies have responsibilities and programs pertaining OSH surveillance. They may serve as major funding sources for OSH surveillance programs. They may also oversight and coordinate OSH surveillance activities. | 4.4 | Y |
|  |  | State agencies | State agencies such as state health departments, labor departments, workers' compensation bureaus/divisions, or employment security departments, play a critical role in collecting, analyzing and disseminating data from local sources and conduct various OSH surveillance systems in partnership with federal agencies and other national and local organizations. | 4.6 | Y |
|  |  | Employers & employees | Employers are asked to participate in the collection and submission of data relevant to their company and their industry, including injuries and illnesses happened to their employees. Employees themselves may choose to (or not to) seek medical care, file a workers' compensation claim, or report poisoning or other work-related conditions. Employers and employees' engagement may directly impact the quality and representativeness of data collected. Employers and employees (e.g. unions) can also be helpful distributors of surveillance findings. | 3.8 | N |
|  |  | Medical care providers | Include clinics/physicians, testing laboratories who make diagnosis and treat health conditions. They are required to report selected diseases and health conditions to state health agencies. Hospitals and emergency departments can provide important medical record data. | 4.2 | Y |
|  |  | Professionals and professional organizations | Provide technical supports. For example, occupational health advisory committee of the surveillance system provides subject matter expertise on surveillance activities and improvement. NGOs, businesses (e.g. Electronic Medical Record developers) and research institutions provide education programs, technological assistance or resources for promoting data dissemination and use. They may also provide data to the surveillance system. | 3.6 | N |
|  |  | Other stakeholders | Include communities and individuals who are affected by/benefited from the surveillance activities, organizations and the general public who are interested in the surveillance and the data it generates. | 3.9 | N |
| Activities | Data Processing | / | The function of system to synthesize and analyze data at individual and aggregated levels and produce interpretable results. Protocols should be in place specifying data collection methods & procedures. | 4.7 | Y |
|  |  | Data collection | The process of collecting and recording data into the system. Different local and state health agencies, medical care providers, insurance companies, businesses and other organizations may be involved in data collection and curation before the surveillance system obtains access to the data. | 4.6 | Y |
|  |  | Case investigation | In case-based surveillance systems, cases need to be investigated for details on the injury/illness/ disease, causing/contributing factors, and other associated factors. | 3.8 | N |
|  |  | Data analysis | Routine data analysis should be established on daily/monthly basis or in longer time frame depending on the surveillance purposes/needs. Also allow analysis "on demand" for more data exploration, more specialized analysis, or dealing with urgent issue. | 4.3 | Y |
|  |  | Data interpretation | Analysis results should be interpreted in the occupational safety and health context to establish relevance. | 4.7 | Y |
|  | Early Detection | / | Timely data processing, analysis and dissemination to guide immediate actions to stop workplace hazards and possible diseases clusters. Early detection may not be a critical function in current OSH surveillance practice. However, there is the need for more timely case detection and investigation to guide effective intervention action. | 3.9 | N |
|  |  | Provide timely data | In current OSH surveillance practice, there could be months to years lag in data incubation. New and emerging data sources such as syndromic data system, electronic records data, social media or other crowd sourcing data may provide hope for more timely data. | 3.5 | N |
|  |  | Detect clusters & unusual events | Clusters or unusual events such as multiple injuries/illness happening in the same workplace at the same time may signal unsafe working condition. More timely data can help to detect clusters and unusual events in a quick manner. | 3.8 | N |
|  |  | Guide immediate actions | More timely data help to guide immediate actions to prevent more events from happening on workers. Data can be provided to relevant organizations if the surveillance system does not have the action capacity. For example, OSHA may use the information to guide workplace inspections on workplace hazards. | 3.5 | N |
|  | Ongoing Monitoring | / | Ongoing surveillance activities with an emphasis on identifying burdens and trends over time and population. | 4.9 | Y |
|  |  | Track burdens & trends | Measure burdens of work-related injuries or illnesses (e.g. frequency, rate, & severity) and monitor trends over time and space. | 4.7 | Y |
|  |  | Identify populations at risk | Identify industries, occupations, and worksites as well as populations, defined by sociodemographic characteristics or work arrangements, at high risk for work-related injury, illness, or hazardous exposures. | 4.7 | Y |
|  |  | Detect workplace hazards | Detect workplace hazards and facilitate the investigation of diseases/events linked to occupational exposures. | 4.8 | Y |
|  | Data Dissemination | / | The function to routinely and actively disseminate data and information to the public to guide actions on improving occupational safety and health. | 4.6 | Y |
|  |  | Produce dissemination materials | Consistently produce data and information for dissemination. Customizing contents and formats relevant to targeted users promotes the use of information for actions by stakeholders. | 4.3 | Y |
|  |  | Dissemination timing | Data dissemination on a routine and timely basis. Also consider flexibility in the plan. | 4.1 | Y |
|  |  | Dissemination channels/mechanisms | Plan dissemination mechanism and maximizing dissemination channels. Wide collaboration with local, state and national government departments, institutions, industry associations and employee unions, and other agencies helps to identify more ways and channels for dissemination. | 4.3 | Y |
|  | Supporting activities | / | Functions that facilitate implementation of the core functions | / | Y |
|  |  | Supervision | Activities of directing and watching over the surveillance work and performance and ensuring the necessary resources and logistics in place. | 3.7 | N |
|  |  | Management | The day to day management of the surveillance system. It ensures that planned activities are implemented according to working schedule and protocol. | 4 | Y |
|  |  | Training | The process to build skills and knowledge for staff and other stakeholders involved within the surveillance system through knowledge transfer and/or hands-on coaching. | 4.3 | Y |
|  |  | Communication & coordination | Effective communication and coordination within and outside the surveillance system are needed to facilitate collaborations and ensure various surveillance activities can be conducted effectively and efficiently. Adequate communication improves mutual understanding on surveillance objectives and requirements. Coordination facilitates integration among different functions and components to achieve cost-effectiveness. | 3.9 | N |
|  |  | Surveillance evaluation | Evaluation of the system periodically and when needed throughout the surveillance system's life cycle. Important to ensure that the planned activities are on track and the surveillance objectives are being achieved. Findings and recommendations resulting from the evaluation should be disseminated and utilized to improve the system. | 4 | Y |
| Outputs | Publications | / | Data (e.g. summary statistics, case investigation, trend analysis, etc.) and interpretable information generated routinely and on demand need to be reported in various forms, depending on the surveillance purposes and dissemination methods. | 4.8 | Y |
|  |  | Technical reports | Describes the process, progress, and/or results of surveillance activities, including data collection, generation and interpretation in a scientific manner. Technical reports may include findings and recommendations. | 4.2 | Y |
|  |  | Media reports | A way to disseminate surveillance findings and information to general audience. Could include multiple media sources, such as newspapers, news reports, webpages, webinars. Social media is promising in reaching out to more end-users. | 4.2 | N |
|  |  | Conference presentations | Way to disseminate surveillance and connect with academic researchers and professionals from various fields, who could potentially be data users and collaborate with the surveillance system. | 4.2 | Y |
|  |  | Peer-reviewed publications | An official way to disseminate important findings to academic researchers, professionals and other interested people. Peer-reviewed publications are important in producing impact. | 4 | Y |
|  |  | Educational Materials | A way to disseminate information to targeted audience, such as employers and employees dealing with work-related hazards, injuries or illnesses. Materials need to be tailored for the reading level of targeted audience in terms of content, format and language used. | 4.5 | Y |
|  | Datasets | / | Individual level and aggregated data records released by the surveillance system to the public and interested organizations/individuals for research and other purposes. | 3.8 | N |
|  | Other outputs | / | Other outputs in the surveillance system may include research and outreach, intervention prevention guidance, strategic policy recommendations, etc., depending on the surveillance system's objectives and resources available. | 3.8 | N |
| Outcomes | Short & mid-term outcomes | Support research | Provides data for hypotheses generation and support applied research & epidemiological studies. | 3.7 | N |
|  |  | Guide intervention programs | Data and information produced help to guide the planning, implementation, and evaluation of actions & programs intended to prevent and control work-related injuries, illnesses, and hazardous workplace exposures. | 4.7 | Y |
|  |  | Increase awareness and knowledge | Data and information produced help to increased awareness and knowledge of occupational safety and health among public health and occupational health professionals, decision makers, and working populations. | 4.5 | Y |
|  | Long-term outcomes | Policy changes at national/state/local level | Surveillance data and findings inform policy-making at different administrative levels, which addresses issues and gaps in occupational safety and health field. | 4.5 | Y |
|  |  | Safer workplace | Results and findings in the surveillance system help to create a safer workplace, where hazardous exposures are eliminated and interventions to promote workers' health are implemented. | 4.9 | Y |
|  |  | Reduced workplace injuries, illnesses & diseases | The ultimate goal and overarching outcome of the surveillance system is to lead reduced work-related injuries, illnesses, and diseases. | 4.9 | Y |

**Table 2-2.** OSH surveillance attributes selected with high/low consensus

| **Logic Model** | **Component** | **Attribute** | **Definition** | **Mean Rating** | **High Consensus** |
| --- | --- | --- | --- | --- | --- |
| Inputs | Infrastructure | Legislative support | Existence of legal and regulatory requirements to support the establishment and maintenance of the surveillance system and its activities. | 3.6 | N |
|  |  | Compliance | Degree to which the surveillance system complies with all relevant legislation, regulations and policies, including ethics and confidentiality requirements. | 4.2 | Y |
|  |  | Sustainability | The system is able to sustain itself with adequate financial, human and material resources. | 4.8 | Y |
|  | Surveillance strategy | Confidentiality | Privacy and data confidentiality requirements for the collection, storage, backup, transport and retrieval of information (especially over the internet), based on relevant standards and guidelines. | 4.5 | Y |
|  |  | Significance | Objectives, priorities and events under surveillance in the system reflect significant occupational safety and health concerns. | 4 | N |
|  |  | Feasibility | Surveillance strategies and methods are suitable and applicable to the surveillance objectives and available resources. | 4.5 | Y |
|  |  | Transparency | Surveillance strategies, methodologies and activities are planned and revised following a well-established, sound and transparent approach and process. | 4.4 | Y |
|  | Stakeholders | Acceptability | Stakeholders understand the purpose of the surveillance system, advocate the system, feel the system is useful, and/or are willing to participate in surveillance activities. | 4.5 | Y |
|  |  | Mutual understanding | Relevant information is shared among stakeholders, including surveillance objectives and requirements (such as privacy and confidentiality requirements), the flow of surveillance data and process, and the methodologies of information dissemination and utilization. This helps to achieve mutual understanding and expectations among stakeholders. | 4.2 | Y |
|  |  | Mutual benefit | The capability of the surveillance system to create opportunities where collaborations can benefit both the system and its partners/collaborators. | 4 | Y |
| Activities | / | Relevance | Degree to which functions and activities in the surveillance system are relevant to its objectives and priorities as per occupational safety and health needs. | 4.3 | Y |
|  |  | Adherence | Degree to which the surveillance system follows guidelines, standards, protocols in its core and supporting functions. | 4.1 | Y |
| Outputs | / | Accessibility | Ability of the surveillance system to make data and information accessible to those who need it and when they need it. | 4.5 | Y |
|  |  | Usability | Data and products produced by the surveillance system should be tailored to meet the needs of intended users. | 4.6 | Y |
| Outcomes | / | Usefulness | Ability of the surveillance system to directly and indirectly contribute to the prevention and control of adverse occupational health conditions and improve workers' safety and health. | 4.7 | Y |
| System-related | / | Simplicity | A guiding principle in designing and implementing the surveillance system, including the organizational structure, the surveillance strategies, information flow from data providers to data users, coordination of various surveillance activities. Simplicity is conductive to the effectiveness and efficiency. All different components in the surveillance system should be designed to avoid unnecessary complexity. | 3.8 | N |
|  |  | Flexibility | Ability of the system to adapt to changes in operating conditions, technologies or information with little additional time, personnel, or funds. | 4.3 | Y |
|  |  | Timeliness | Capability of the system to finish working steps and fulfills its objectives with a speed that's quick and appropriate. | 4.2 | Y |
|  |  | Stability | Ability of the surveillance system to be operational and provide surveillance products reliably and stably (without failure). | 4.5 | Y |
|  |  | Integration | Ability of the surveillance system or components in the system to integrate/connect with other surveillance or public health systems to enhance interoperability, effectiveness and/or to reduce cost. | 3.3 | N |
|  |  | Effectiveness | Ability of a surveillance system to achieve its intended objectives. | 4.5 | Y |
|  |  | Cost-effectiveness | Goes beyond effectiveness by bringing in a reference to the amount of resources involved. A surveillance system is cost effective if it identifies data with the biggest impact on improving worker safety and health while minimizing the cost of collecting the data. Cost-effectiveness analysis should consider the societal costs associated with the occurrence of events under surveillance, i.e., the costs associated with the health and productivity consequences of occupational exposures, injuries, illnesses, and mortality on workers, their families, and society, including both direct and indirect costs. | 3.6 | N |
| Data quality | / | Validity | Degree to which information correctly describe the health event it was designed to measure. | 4.3 | Y |
|  |  | Sensitivity | The ability of the surveillance system to capture true cases/events or outbreaks/clusters. | 4.1 | Y |
|  |  | Specificity | The proportion of individuals not having the occupational health related event identified by the system as not having the event. | 4.1 | Y |
|  |  | Predictive Value Positive (PVP) | The proportion of reported cases/events that are actually true cases/events. | 4.1 | Y |
|  |  | Representativeness | The extent to which data adequately represent the population under surveillance and relevant sub-populations by time, place, population demographics and socio-demographics. | 4.6 | Y |
|  |  | Consistency | Data have the same meanings (e.g. case definition, diagnosis standards) to allow for consistent interpretation. This includes internal consistency (within a dataset) and external consistency (across different data sources), as well as consistency over time. | 4.5 | Y |
|  |  | Completeness | Required variables recorded in the surveillance datasets are complete without missing data. | 4.1 | Y |
|  |  | Accuracy | Data, statistics, and information produced in the surveillance datasets are accurate without errors. | 4.7 | Y |
|  |  | Clarity | Data, statistics, and information is coded/presented clearly without ambiguity. For example, variables are named appropriately. Data dictionary is clear and easy to follow. | 4.2 | Y |

**Table 2-3.** Example measures selected with high/low consensus

| **Logic Model** | **Component** | **Attribute** | **Measure** | **Mean Rating** | **High Consensus** |
| --- | --- | --- | --- | --- | --- |
| Inputs | Infrastructure | Legislative support | Are there mandatory requirements on the establishment of the system? | 3.4 | N |
|  |  |  | Are there mandatory requirements on data reporting/collection for the events/cases under surveillance? | 4.1 | Y |
|  |  | Compliance | Is the system in compliance with all legal and regulatory requirements? | 3.3 | N |
|  |  | Sustainability | Is funding secure for short-term (e.g. 3-5 years) and long-term (e.g. more than 5 years) future? | 4.8 | Y |
|  |  |  | Are the necessary human resources (e.g. staffing) sustainable for the short-term and long-term future? | 4.8 | Y |
|  |  |  | Are the necessary material resources (e.g. technical infrastructure) sustainable for the short-term and long-term future? | 4.4 | Y |
|  |  |  | Can the system maintain and expand collaborations in the future (e.g. for data collection, analysis and dissemination)? | 4 | Y |
|  |  |  | Can financial, human and material resources support the system to adapt to envisioned changes and/or to expand activities? | 3.1 | N |
|  | Surveillance strategy | Confidentiality | Are privacy and confidentiality requirements clearly specified in protocols/documentation? | 4.3 | Y |
|  |  |  | Are the requirements reviewed regularly to be updated as necessary? | 3.5 | N |
|  |  |  | Are well-established methodologies in place to secure confidentiality in the data collection and transport, especially over the internet? | 4.1 | Y |
|  |  |  | Is there a process to check for and document deviations and corrective actions when privacy or confidentiality is breached? | 4.5 | Y |
|  |  | Significance | Does the system identify significant OSH issues (i.e. potentially catastrophic, associated with big economic and/or social impacts, or concerns among the public or industries)? | 4.4 | Y |
|  |  |  | Once identified, is there a process to set objectives and prioritize events to surveil? e.g. with advisory board or stakeholders, through consensus, through grant peer review. | 3.5 | N |
|  |  |  | How does the system adjust its objectives and priorities based on emerging OSH issues? | 3.6 | N |
|  |  |  | Are there past examples of the system reflecting significant occupational safety and health concerns? | 3 | N |
|  |  |  | Are populations at risk (defined by age, ethnicity, size, spatial distribution, etc.) identified and tracked in the system on an ongoing basis? | 4.3 | Y |
|  |  | Feasibility | Do the system strategies correspond to objectives and priorities? | 4 | Y |
|  |  |  | Do necessary outside resources exist (e.g. steering committee, technical committee, government affiliation)? | 3.6 | N |
|  |  |  | Do the staff have the required knowledge, skills and experience to operate and maintain the system? Multidisciplinary expertise may be needed for modern day surveillance. | 4.3 | Y |
|  |  |  | Are the surveillance events/cases trackable over time given the data sources available? | 4 | Y |
|  |  |  | Do the resources available match the strategies and processes (data collection, data analysis, and data dissemination, etc.)? | 4.5 | Y |
|  |  | Transparency | Are important details (e.g. surveillance strategies, planned activities, methodologies for data collection and analysis, changes) clearly documented in system protocols/documentation on a regular basis? | 4.3 | Y |
|  | Stakeholders | Acceptability | Are stakeholders aware of the existence of legal and mandatory requirements for the surveillance activities? | 3.8 | N |
|  |  |  | Do stakeholders actively communicate with the system staff? | 3.8 | N |
|  |  |  | Participation rate/responding speed of stakeholders in a certain surveillance activity (e.g. meeting, data request, survey). | 3.5 | N |
|  |  |  | The level of willingness of stakeholders to collaborate with the surveillance system. | 4 | Y |
|  |  | Mutual understanding | Are responsibilities clearly specified for staff and collaborators in the surveillance system? | 3.4 | N |
|  |  |  | Is there a mutual understanding of security, confidentiality and privacy process between the system and stakeholders (e.g. data providers, data users)? | 4 | Y |
|  |  |  | Has a trusted relationship been established between the surveillance system and its collaborators? | 3.4 | N |
|  |  | Mutual benefit | Are opportunities created for collaborators to benefit from the system? | 3.4 | N |
| Activities | / | Relevance | Is there an up-to-date logic model/flowchart that links the system's functions and objectives? | 3.3 | N |
|  |  |  | Is each function/activity necessary to achieve objective/meet priorities? | 3.3 | N |
|  |  |  | Are performance indicators specified in the surveillance system? Example indicators include expected frequency of case reporting, data analysis and dissemination, or frequency of work meetings? | 3.8 | N |
|  |  | Adherence | Is there auditing process to track how function protocols are followed? | 3 | N |
|  |  |  | Are methods established for data collection and processing to ensure high-quality data? | 4.4 | Y |
| Outputs | / | Accessibility | Is there an appropriate plan for dissemination to increase accessibility, including channels and formats? | 4.1 | Y |
|  |  |  | Is the system able to respond to internal and external data requests? Balance between confidentiality requirements and benefits of feeding data to researchers and the public may be a challenge. | 4 | Y |
|  |  | Usability | Are useful data interpretations provided to the audience beyond the data (e.g. statistics)? | 4.3 | Y |
|  |  |  | Are products tailored to the need of the end users (e.g. reading level, knowledge level, language, industry/occupation, disability needs, culture and formats of communication)? | 4 | N |
|  |  |  | Can statistics be stratified by factors of interest, such as sex, age, ethnicity, socioeconomic status, geography, etc? | 4.4 | Y |
|  |  |  | Does the system seek feedback on usability by stakeholders to guide improvement? | 3.6 | N |
| Outcomes | / | Usefulness | Does the system provide timely data to detect outbreaks/clusters/unusual events? Calculate the number/percentage of events detected. | 4.3 | Y |
|  |  |  | Does the surveillance data allow for investigation of clusters, outbreaks, and/or unusual events to guide timely mitigation action? Calculate the number/percentage of the investigated/actionable. | 4.1 | Y |
|  |  |  | Does the system measure the burden of work-related injuries or illnesses and monitor trends over time and space? | 4.6 | Y |
|  |  |  | Can the surveillance data guide identification of worker populations at risk? | 4.5 | Y |
|  |  |  | Does system track leading indicators of occupational safety and health (e.g. workplace hazards)? | 4.1 | N |
|  |  |  | Can the system detect new/emerging diseases and/or workplace hazards? | 4.3 | Y |
|  |  |  | Has the data dissemination helped to increase the workers' and employers' safety and health awareness? | 4.3 | Y |
|  |  |  | Does the system provide data to support epidemiological studies and applied research/practices? | 3.8 | N |
|  |  |  | Can surveillance data guide the planning, implementation, and evaluation of interventions or programs intended to prevent and control work-related injuries, illnesses, and diseases? | 4.3 | Y |
|  |  |  | Has the surveillance data lead to policy/regulation changes at national/state/local level? | 3.9 | N |
| System-related | / | Simplicity | Is the case definition of events/conditions under surveillance simple and easy to understand? | 3.1 | N |
|  |  |  | Does the system rely on sophisticated data collection methods, multiple data sources and/or multiple collaborators? | 4 | Y |
|  |  |  | Does the system involve active surveillance activities? | 3.1 | N |
|  |  |  | Are system protocols easy to follow? | 4.1 | Y |
|  |  |  | Is the organizational structure simple but sufficiently effective to meet the surveillance needs? | 3.8 | N |
|  |  |  | Does the system use accepted standards for data coding and data sharing so that merging and comparison within/outside the system is easy? | 3.9 | N |
|  |  |  | Are processes and platforms (e.g. software, database) for data reporting, entry, coding, merging, and sharing streamlined and easy to use? | 3.8 | N |
|  |  |  | Is there any part of the system unnecessarily complicated? Is there a better way to streamline the system? | 3.5 | N |
|  |  | Flexibility | Can the system respond to event/case definition changes? | 4.1 | Y |
|  |  |  | Can the system respond to changes and/or variations across existing data sources? | 4.1 | Y |
|  |  |  | Can the system easily incorporate new data sources? | 4 | Y |
|  |  |  | Can the system respond to new data standards (e.g. ICD-9 to ICD-10)? | 4.3 | Y |
|  |  |  | Can the system respond to changing surveillance technologies (e.g. online data sharing, machine learning techniques for data coding)? | 4.3 | Y |
|  |  |  | Can the system adjust surveillance objectives and strategies as needed (e.g. respond to new hazards/conditions)? | 4.4 | Y |
|  |  |  | Is there funding/personnel/material redundancy to support flexibility? | 3.5 | N |
|  |  | Timeliness | Time required for collaborating agencies to process data before it is transferred to the system. | 4.1 | Y |
|  |  |  | Time needed to investigate and analyze the data for mortality/morbidity, trends, outbreak, risk factors. | 3.8 | N |
|  |  |  | Time needed for the surveillance system to release warning alerts (outbreak, unusual events) and/or data/statistics reports. | 4 | Y |
|  |  |  | Time needed for the system to respond to requests (e.g. data request, regular/urgent work needs). | 3.5 | N |
|  |  |  | Overall time spent from events (exposure, seeking health information, medical care, and/or medicine) to data dissemination and eventually to public health actions. | 3.5 | N |
|  |  |  | Are the time spent appropriate for the objectives of the system? | 3.4 | N |
|  |  | Stability | Can the system continue its planned activities with staff turnover or other resource problems? | 4 | Y |
|  |  |  | Are data sources and data collection collaboration stable to ensure ongoing surveillance? | 4.3 | Y |
|  |  |  | Can the system maintain its activities under changing situations such as coding system changes, technological updating, switching technical platforms? | 4 | Y |
|  |  | Integration | Is the system connected with other public health systems within or outside the same organization (in terms of data collection/processing/sharing)? | 3.4 | N |
|  |  |  | Does the system follow standardized data collection and sharing for integration ease? | 3.6 | N |
|  |  |  | Are problems/opportunities identified with functions as they relate to each other? | 3.1 | N |
|  |  |  | Are potential issues addressed for a successful integration (e.g. to avoid annexation)? | 3.1 | N |
|  |  |  | Are there data sharing mechanisms among different surveillance functions, which includes information to share, to whom the information being communicated, and data sharing platform? | 3.5 | N |
|  |  | Effectiveness | Is the system effective in accomplishing its stated objectives? | 4.6 | Y |
|  |  |  | Are various methods used to increase effectiveness, such as shared efforts on data collection/processing/dissemination through internal and external collaboration? | 3.6 | N |
|  |  |  | Percentage of planned tasks (e.g. obtaining source data, outbreak detection & case investigation, data analysis, producing data reports) accomplished by due dates. | 4 | Y |
|  |  |  | What are the costs (fixed and variable) associated with each surveillance activity in the system? | 3.5 | N |
|  |  | Cost-effectiveness | What are the costs (direct and indirect, individual and societal) for injuries/illnesses/events under surveillance? | 3.6 | N |
|  |  |  | Is cost-effectiveness (financial and social) a consideration in decision-making for objectives and priorities, and the selection of surveillance methodologies? | 3 | N |
|  |  |  | Is there up-to-date cost-effectiveness analysis done for the system? | 3 | N |
| Data quality |  | Validity | Is their standard scientific evidence that the event under surveillance is work-related, or significantly associated with occupational factors. | 4.1 | Y |
|  |  |  | Validity of the survey constructs. | 4.3 | Y |
|  |  |  | Are the data of sufficient quality to generate a valid estimate? | 4.5 | Y |
|  |  |  | Are biases introduced in data collection process (e.g. case reporting, data coding, method to identify work-relatedness) that affect the validity of measurements? | 4.1 | Y |
|  |  | Sensitivity | The likelihood/percentage that workers will seek medical care or compensation and be reported/captured to the surveillance system? | 3.4 | N |
|  |  |  | Sensitivity of the screening/diagnosis procedure. | 4 | Y |
|  |  |  | The proportion/number of cases/events are captured by the surveillance system compared to certain standard OSH data source(s)? | 3.5 | N |
|  |  |  | Is there any active approach used to help to detect true cases/events? | 3.5 | N |
|  |  |  | Can the system accurately detect clusters, outbreaks or usual events in an appropriate time frame? | 3.4 | N |
|  |  | Specificity | Specificity of the screening/diagnosis procedure. | 3.3 | N |
|  |  |  | The proportion/number of non-cases/events correctly determined by the surveillance methodology/algorithm (e.g. by comparing two methodologies identifying work-related cases). | 3.3 | N |
|  |  | Predictive Value Positive (PVP) | The proportion/number of cases/events captured by the surveillance system that are confirmed as true cases/events. | 3.9 | N |
|  |  |  | Is there active approach used to verify cases/events? | 4 | Y |
|  |  | Representativeness | Does the system reflect the characteristics of working populations under surveillance? | 4.1 | Y |
|  |  |  | Does the system reflect the change of population characteristics over time? | 4.1 | Y |
|  |  |  | Are there sub-populations of interest excluded or under-reported? | 4.3 | Y |
|  |  | Consistency | Are data collected by different entities within the system following consistent and rigorous formats? | 4 | Y |
|  |  |  | Are data collected from different sources share common methodology (e.g. case definition, coding systems, and classifications)? | 4.3 | Y |
|  |  |  | Is data coded the same way over time to track trends? | 4.4 | Y |
|  |  | Completeness | Proportion of missing or blank data? | 4 | Y |
|  |  |  | What is the reason for missing data? | 3.8 | N |
|  |  |  | What is the impact of missing data? | 4.1 | Y |
|  |  | Accuracy | Are there proper quality control measures to identify and correct errors in place? | 4.5 | Y |
|  |  |  | Is there data corruption due to incorrect data merging or conversion? | 4.1 | Y |
|  |  | Clarity | Are variables coded clearly and consistently? | 4.5 | Y |
|  |  |  | Does the surveillance system keep a clear and easy-to-follow, up-to-date data dictionary? | 4.4 | Y |
|  |  |  | Are data provided by the system in formats easy to understand and provided with accurate data dictionaries? | 4 | Y |
|  |  |  | Are educational materials and other materials for dissemination formatted clearly and precisely? | 3.4 | N |
